# Household dysfunction and dating violence perpetration: the moderating effects of parental monitoring and closeness among middle school adolescents in Southeast Texas

**DOI:** 10.1186/s12889-025-24549-4

**Published:** 2025-10-03

**Authors:** Sumaita Choudhury, Emily T. Hébert, Timothy J. Walker, Christine M. Markham, Ross Shegog, Robert C. Addy, Melissa F. Peskin

**Affiliations:** 1 The University of Texas Health Science Center at Houston (UTHealth Houston) School of Public Health, 7000 Fannin Street, Houston, TX 77030 USA; 2 The University of Texas Health Science Center at Houston (UTHealth Houston) School of Public Health, Austin location, 1616 Guadalupe, Austin, TX 78701 USA

**Keywords:** Household dysfunction, Dating violence, Parental monitoring, Parental closeness, Adolescents

## Abstract

**Background:**

Adolescent dating violence (DV) perpetration is a significant public health issue affecting millions of adolescents in the United States. Adverse childhood experiences such as household dysfunction (HD) have been exhibited to be risk factors for DV perpetration during adolescence and adulthood. Furthermore, the literature is scant regarding how positive parent-child relationships can mitigate these risk factors and decrease HD-related consequences among sixth-grade adolescents. Thus, our study examined the moderating effects of parental monitoring and closeness regarding the association between HD and DV perpetration among middle schoolers in Southeast Texas.

**Methods:**

In our cross-sectional study, we used the baseline data from a DV prevention intervention program for sixth graders aged 11–14 years (*N* = 101), Me & You Tech. We conducted adjusted multivariable regression models to examine the link between HD and DV perpetration, along with the moderating effects of parental monitoring and closeness.

**Results:**

The multivariable logistic regression results regarding the association between lifetime DV perpetration, HD, parental monitoring, parental closeness, and covariates demonstrated a significant association between HD and lifetime DV perpetration (AOR = 1.36; 95% CI: 1.01, 1.83); however, the moderators did not exhibit significant effects.

**Conclusions:**

Our study highlights how early exposure to HD can lead to violent behaviors. Future research should investigate parental protective factors to enhance early prevention of HD-related consequences such as DV perpetration.

**Trial number:**

NCT05225727.

**Trial registration date:**

January 24, 2022.

**Trial registration:**

https://classic.clinicaltrials.gov/ct2/show/NCT05225727.

**Supplementary Information:**

The online version contains supplementary material available at 10.1186/s12889-025-24549-4.

## Background

Adolescent dating violence (DV) can include physical DV, sexual DV, psychological DV (i.e., verbal, relational, digital/cyber, and threatening DV), and stalking among adolescents in a dating or romantic relationship [1]. DV has been associated with poor mental and physical health outcomes such as depression, substance use disorders, and antisocial behaviors among adolescents [1, 2]. According to the Centers for Disease Control and Prevention’s (CDC) 2021 Youth Risk Behavior Surveillance System (YRBS) Survey, about 8.5% of adolescents who dated in the past year reported physical DV, 9.7% reported sexual DV, and 13.6% reported either or both [3, 4]. 

Adverse childhood experiences (ACEs) are traumatic events that individuals experience during their childhood before age 18, including physical and sexual abuse, physical and emotional neglect, and household dysfunction (HD) [5]. ACEs are risk factors for DV perpetration among adolescents [6]. HD, a domain of ACE, is defined as children growing up in an environment, where they witness and/or experience domestic violence, parental divorce, food insecurity, homelessness, household member history with substance use, disability, incarceration, death, or mental health disorders [5, 7–9]. An increasing body of literature has linked HD with DV perpetration among adolescents, which has been shown to have long-term consequences regarding maintaining healthy relationships both during adolescence and adulthood [10–16]. As many adolescents start their romantic relationships during middle school, particularly during sixth grade [16, 17], it is imperative to study middle school-aged adolescents (aged 11–14 years) to examine the impact of early HD exposures on DV perpetration to help intervene early and inform efforts for strategic interventions to prevent long-term HD and DV-related consequences.

Protective factors such as positive relationships with parents/caregivers (i.e., parental monitoring and parental closeness) have shown to mitigate the negative effects of ACEs along with decreasing DV perpetration and DV victimization among adolescents [6, 18–20]. Parental monitoring captures adolescents’ perceptions regarding their parent/caregiver’s knowledge about their personal lives, and parental closeness encompasses the attachment, trust, and interconnectedness adolescents have with their parent/caregiver [17, 21–23]. Parental monitoring and parental closeness or support from parents/caregivers have also shown to decrease ACEs and DV among adolescents [6]. Furthermore, positive relationships with parents, schools, and communities could also serve as coping strategies to decrease the consequences of ACEs and related problematic or violent behaviors among adolescents [24]. In contrast, adolescents who did not have protective factors were more prone to poor physical and mental health and were more likely to engage in delinquent behaviors [25]. 

To date, studies that examined the role of protective factors regarding ACEs and violent behaviors among adolescents assessed different types of parental and familial protective factors with varying definitions [6, 19, 20, 26]. For example, Gorman-Smith et al. [26] assessed how family functioning (i.e., family beliefs, family cohesion, parenting practices, and discipline) moderated the relation between exposure to community violence and violence perpetration among minority male youth. They found that compared to those with better family functioning, youth from struggling families with poor parenting practices exhibited emotional dysregulation from community violence exposure and later engaged in violence. [26] However, this study did not assess DV perpetration as an outcome nor HD as the primary ACE exposure. Furthermore, the family functioning protective factor examined in this study did not include parental monitoring or closeness. [26]

Other studies that evaluated the roles of positive relationships with parents (i.e., parental closeness or support and parental monitoring), peers (i.e., supportive friendships and social support), and schools (i.e., school belonging and academic achievement) among adolescents have either focused on how they influence the relation between ACEs and dating violence victimization [19] or how the factors moderated the trajectories between exposure to community violence and DV perpetration from middle school to high school [6]. However, none of those studies examined how parental monitoring and closeness individually moderate the association between HD and DV perpetration among sixth grade middle schoolers. It is important to specifically assess these two factors as moderators of the association between HD and DV perpetration because previous studies that have examined protective factors assessed different aspects of parental/adolescent relationships that did not adequately capture parental monitoring and closeness. Increased parental monitoring and closeness may reduce the impact of HD on adolescents through mechanisms like fostering emotional security, attachment, trust, and building resilience, ultimately decreasing their likelihood of committing DV perpetration during adolescence [27, 28]. Furthermore, examining these parental factors separately will help inform parent-focused interventions to decrease the immediate and long-term negative impact of HD and DV perpetration.

Thus, it is vital to understand the individual roles of these parental protective factors regarding the association between HD and DV perpetration among middle school adolescents as an essential criterion for early intervention initiatives. Consequently, the purpose of our study was to examine the moderating effects of parental monitoring and closeness regarding the association between HD and DV perpetration among middle schoolers aged 11–14 years in Southeast Texas. We hypothesized that higher levels of parental monitoring and closeness would alleviate the positive association between HD and DV perpetration, suggesting a protective role of supportive parenting in early adolescence.

## Methods

### Parent study

#### Intervention overview

Me and You (MY) Tech: A Socio-Ecological Solution to Teen Dating Violence for the Digital Age is an internet-based dating violence prevention program developed for sixth-grade middle school students aged 11 to 14 years in Southeast Texas, adapted from the Me & You DV intervention [16, 29]. 

#### Study design

The MYTech intervention was evaluated in a randomized two-arm design study, including four middle schools [30]. Two schools were randomly assigned to receive the curriculum, and two schools were randomly assigned to receive standard care, including health education knowledge from the state textbook. Outcomes were evaluated at baseline, three months following intervention completion, and nine months after baseline.

#### Recruitment and study procedure

During classroom sessions, research personnel described the study purpose and enrollment criteria to all eligible students. Project staff also conducted active, onsite recruitment by providing information directly to students during class. Informational materials were sent home, and all parental consent materials were translated to accommodate diverse linguistic needs. All student and parental consent and assent forms were obtained before study participation. A school site coordinator was identified for each school and received a small stipend to support recruitment efforts. Students received a $5 incentive for completing both the baseline and three-month follow-up surveys, and a $10 incentive for completing the nine-month follow-up survey.

A total of 794 adolescents were initially approached to participate in the study. Of these, 280 adolescents returned a parent permission form. Among those, 189 received parental permission to participate, and 134 adolescents provided assent. Of the 134 adolescents who provided assent, 126 adolescents completed the full baseline MyTech survey. This relatively low response rate reflects the challenges of recruitment during the COVID-19 pandemic and introduces the possibility of selection bias. Therefore, results should be interpreted with consideration of potential differences between participants who enrolled and those who did not complete the survey or provided incomplete data.

#### Ethics approval and consent to participate

This research study was conducted in accordance with the Declaration of Helsinki. The Institution Ethics Review Board at the University of Texas Health Science Center at Houston approved the study (HSC-SPH-19-0253, May 02, 2019). Informed consent was obtained from the parents or legal guardians of all participants under the age of 16, and student assent forms were obtained before the dissemination of the baseline surveys.

#### Present study design, setting, and data collection

We used the baseline data (collected from March 2023 to April 2023) of the MYTech program. We conducted a cross-sectional analysis to assess whether protective factors such as parental monitoring and parental closeness moderate the association between HD and DV perpetration among middle school adolescents.

#### Eligibility criteria

Eligible participants for the MYTech study included adolescents aged 11 to 14, currently in sixth grade, enrolled at a participating middle school.

### Measures

#### Dependent variable

##### DV perpetration

The Conflict in Adolescent Dating and Relation Inventory (CADRI) DV perpetration subscales (Wolfe et al., 2001) were used to assess physical, psychological, threatening, and sexual DV perpetration. Digital DV perpetration items were adapted from Zweig et al. [31] and Picard [32]. Physical DV (α = .62) was assessed by four items (i.e., “I threw something at him/her.”); psychological DV (α = .81) was assessed by 13 items (e.g., “I said things just to make him/her angry”); threatening DV (α = .80) was measured by four items (e.g., “I tried to frighten him/her on purpose”); sexual DV was measured by one item (e.g., “I kissed him/her when he/she didn’t want me to”); and digital DV (α = .89) was measured by 12 items (e.g., “I posted embarrassing photos or other images of him/her online”) with dichotomized response options“yes” or “no” [16, 33] (Supplement 1). These measures have been used previously among middle school adolescents.^16 ^

A lifetime DV perpetration composite variable was created to collapse DV perpetration response items across the CADRI subscales and digital DV measure. Adolescents who participated in one or more DV perpetration behaviors related to physical, threatening, sexual, or digital and/or reported three or more indications for perpetrating psychological DV were categorized as perpetrators and were coded as “yes.” [16] Adolescents who reported ever having a dating partner (i.e., having a boyfriend or girlfriend; romantically involved with someone) but did not commit DV perpetration, or adolescents who never dated, or did not have dating partners in the past 12 months, were classified as non-perpetrators and coded as “no.” [16]

#### Independent variable

##### Household dysfunction

The newly developed age-appropriate HD measure was created using established methods and has evidence of validity [34]. The HD measure assessed adolescents' exposures to parental separation/divorce, witnessing interparental violence, household member(s) with mental health and/or substance use disorder, household member(s) with a disability, family history of incarceration, death in the family, housing instability and household food insecurity, using a newly-developed age-appropriate 10-item measure with dichotomized response options “yes” or “no” [34] (Supplement 2). We used cumulative scores for HD by summing the total number of HD exposures per study participant with a score ranging from 0 to 10, with 0 being no exposure to HD and 10 being extreme exposure to HD [35–38].

#### Moderating variables

##### Parental monitoring

Parental monitoring (α = .87) evaluated adolescents’ perception of their parental/caregiver’s knowledge about participants’ friends, knowledge regarding participants’ after-school activities, how participants spend money, and more, using five items on a 4-point Likert Scale (1 = don’t know much, 2 = know a little, 3 = know a moderate amount, and 4 = know a lot) (Supplement 3). The mean score of parental monitoring was calculated for each participant, with the average item score ranging from 1.00 (low) to 4.00 (high) [21] with higher scores indicating greater parental monitoring. 

##### Parental closeness

Parental closeness (α = .92) assessed adolescents’ attachment, trust, and interconnectedness with their parent/caregiver using six items with a 5-point Likert Scale (1 = strongly disagree, 2 = disagree, 3 = neither agree nor disagree, 4 = agree, and 5 = strongly agree). These items included questions pertaining to adolescents’ perceptions regarding their parents/caregivers' interests in knowing about their involvement in school, parental affection, and more (Supplement 4). We calculated composite scores of parental closeness for each participant, ranging from 6-30 with higher scores indicating greater parental closeness [17, 22, 23].

##### Covariates

Based on empirical evidence and theoretical significance [16, 39, 40], the current study adjusted for the following covariates: age (11 years old to 14 years old), gender identity (girl, boy, or prefer to self-describe), race and ethnicity combined into a composite variable and recoded into three categories (African American, Hispanic, and Another Race (which included American Indian or Alaskan Native, Native Hawaiian or Other Pacific Islander, Asian, Multiracial or mixed race, White or Other)), and sexual orientation recoded into a dichotomized variable (gay/lesbian/bisexual/other or heterosexual). 

### Statistical analysis

We used STATA/BE software 17.0 to conduct all statistical analyses [41]. A total of 126 adolescents completed the MyTech baseline survey. However, we restricted our analytic sample (N =101) to adolescents who provided complete responses for all variables assessed in the study. First, we examined the descriptive statistics of the study sample’s baseline characteristics. We reported mean and standard deviation for the continuous variable and frequencies and percentages for categorical variables. 

Second, we conducted bivariate analyses to assess the independent associations of HD, parental monitoring, parental closeness, and sociodemographic factors with DV perpetration. We used independent t-tests and Pearson chi-squared tests as appropriate to identify significant differences among sociodemographic factors, HD, and parental protective factors among adolescents who engaged in DV perpetration versus non-participation in DV perpetration. Third, we employed three separate adjusted multivariable logistic regression models to examine the association between HD and DV and the effects of parental monitoring and parental closeness. In Model 1, we assessed the association between HD and DV perpetration while adjusting for parental monitoring, parental closeness, and covariates. In Model 2, we employed a moderation analysis to examine whether the effects of HD on DV perpetration vary by parental monitoring while adjusting for parental closeness and covariates. For Model 3, we performed a moderation analysis to evaluate whether the effects of HD on DV perpetration vary by parental closeness while adjusting for parental monitoring and the covariates. Fourth, we assessed the Intraclass Correlation Coefficients (ICCs) for possible clustering effects but found them to be below 0.01. Finally, multicollinearity was assessed using the mean Variance Inflation Factor (VIF), which was approximately 10.82. Despite slightly exceeding the threshold of 10, all variables were retained due to their theoretical importance and to preserve critical information for addressing our study purpose. 

Furthermore, this study utilized data from MYTech, which was designed to examine the effectiveness of an online healthy relationships curriculum to reduce DV perpetration among middle schoolers in Southeast Texas. The inclusion of the interaction term in the analysis was exploratory and based on a secondary research question. As the study was not powered for this specific analysis, the results of the interaction term analysis should be interpreted with caution, and replication in larger, adequately powered samples is recommended to confirm these findings. Findings from this exploratory analysis underscore the importance of future research with larger, adequately powered samples to further examine these associations.

## Results

Overall, the mean age of our analytic sample was 11.80 (±0.65) years (Table 1). Approximately, 54.46% of our sample identified as girls, 57.43% of adolescents reported being Hispanic, and 74.26% self-reported as heterosexuals. The average HD score among our analytic sample was 1.51 (±1.59), with an overall HD score range of 0 – 7. Our sample had a mean parental monitoring score of 2.82 (±0.09), ranging from 1.0 to 4.0. The mean parental closeness score was 20.65 (±6.86), with a summative score range of 6 to 30.

The bivariate results revealed that adolescents who reported lifetime DV perpetration reported an average HD score of 2.14 (±2.17), which was significantly higher than the average HD score of 1.27 (±1.23) among adolescents who did not report lifetime DV perpetration (p<0.05). In addition, adolescents who participated in lifetime DV perpetration reported average parental monitoring score of 2.56 (±0.81), which was marginally lower than the average parental monitoring score of 2.92 (±0.88) among adolescents who did not report lifetime DV perpetration (p=0.067). No other significant differences were found for parental closeness, and the covariates.

**Table 1:** Descriptive statistics and bivariate analyses (*N* = 101)

Variables	Overall Sample *n* (%)	DV Perpetration		No DV Perpetration			*p*-value
		*n*	%	*n*	%		
Gender						0.094	
Girl	55 (54.46)	19	67.86	36	49.32		
Boy	46 (45.54)	9	32.14	37	50.68		
Race/Ethnicity						0.962	
African American	30 (29.70)	8	28.57	22	30.14		
Hispanic	58 (57.43)	16	57.14	42	57.53		
Another Race	13 (12.87)	4	14.29	9	12.33		
Sexual Orientation						0.156	
Gay/Lesbian/Bisexual/Other	26 (25.74)	10	35.71	16	21.92		
Heterosexual	75 (74.26)	18	64.29	57	78.08		
	**M (S.D.)**	**M**	**S.D.**	**M**	**S.D.**		
Age	11.80 (0.65)	11.93	0.66	11.75	0.64	0.226	
Household Dysfunction	1.51 (1.59)	2.14	2.17	1.27	1.23	0.013	
Parental Monitoring	2.82 (0.09)	2.56	0.81	2.92	0.88	0.067	
Parental Closeness	20. 65 (6.86)	19.82	7.42	20.97	6.66	0.453	

*M* Mean, *S.D* Standard deviation, *n * Number of individuals, *DV * Dating Violence

*Gender also included a third category labeled as “prefer to self-describe,” which received zero responses

*Another Race under race/ethnicity category includes (American Indian or Alaskan Native, Native Hawaiian or Other Pacific Islander, Asian, Multiracial, or mixed race, White or Other)

*Other under sexual orientation category includes (not sure yet and something else)

*Household dysfunction was calculated in a summative manner and our sample had a score range of (0–7)

*Parental monitoring was determined by calculating the mean score and our sample had an average score range of (1.0–4.0)

*Parental closeness was calculated in a summative manner and our sample had a score range of (6–30)

### Multivariable regression findings

The multivariable logistic regression results regarding the association between HD, lifetime DV perpetrations, parental monitoring, parental closeness, and covariates demonstrated a significant association between HD and lifetime DV perpetration (AOR=1.36; 95% CI: 1.01, 1.83) (Table 2). No other significant associations were found.

**Table 2:** Multivariable logistic regression analysis of household dysfunction and lifetime DV perpetration, interaction effects of parental closeness with HD, and covariates (*N* = 101)

	DV Perpetration		
	AOR	95% CI	*p*-value
Household Dysfunction	1.32	0.50, 3.46	0.571
Parental Monitoring	0.47	0.19, 1.17	0.104
Parental Closeness	1.04	0.90, 1.20	0.613
Parental Closeness x Household Dysfunction	1.00	0.95, 1.05	0.953

Sociodemographics			
Age	1.64	0.79, 3.41	0.183
Gender			
Girl	Ref.	Ref.	Ref.
Boy	0.59	0.20, 1.71	0.329
Race/Ethnicity			
African American	Ref.	Ref.	Ref.
Hispanic	1.13	0.36, 3.56	0.829
Another Race	0.87	0.18, 4.22	0.862
Sexual Orientation			
Gay/Lesbian/Bisexual/Other	Ref.	Ref.	Ref.
Heterosexual	0.82	0.27, 2.51	0.728

*DV *Dating Violence, *AOR * Adjusted odds ratio, *95% CI * 95% confidence interval, *Ref* Reference group

### Moderation effect of parental monitoring and parental closeness

The multivariable regression analysis showed no significant association between lifetime DV perpetration, HD, parental monitoring, parental closeness, covariates, and the interaction of parental monitoring with HD among sixth graders (Table 3). Additionally, parental closeness did not significantly moderate the association between these variables (Table 4).

**Table 3:** Multivariable logistic regression analysis of household dysfunction and lifetime dating violence perpetration, parental monitoring, parental closeness, and covariates (*N* = 101)

	DV Perpetration		
	AOR	95% CI	*p*-value
Household Dysfunction	1.36	1.01, 1.83	0.045
Parental Monitoring	0.47	0.20, 1.11	0.084
Parental Closeness	1.04	0.94, 1.16	0.465

Sociodemographics			
Age	1.64	0.79, 3.41	0.183
Gender			
Girl	Ref.	Ref.	Ref.
Boy	0.58	0.20, 1.66	0.313
Race/Ethnicity			
African American	Ref.	Ref.	Ref.
Hispanic	1.13	0.36, 3.55	0.830
Another Race	0.87	0.18, 4.21	0.866
Sexual Orientation			
Gay/Lesbian/Bisexual/Other	Ref.	Ref.	Ref.
Heterosexual	0.82	0.27, 2.48	0.198

*DV *Dating Violence, *AOR * Adjusted odds ratio, *95% CI * 95% confidence interval, *Ref* Reference group

**Table 4:** Multivariable logistic regression analysis of household dysfunction and lifetime dating violence perpetration, interaction effects of parental monitoring with HD, and covariates (*N* = 101)

	DV Perpetration		
	AOR	95% CI	*p*-value
Household Dysfunction	2.78	0.66, 11.69	0.162
Parental Closeness	1.03	0.93, 1.15	0.548
Parental Monitoring	0.66	0.23, 1.94	0.455
Parental Monitoring x Household Dysfunction	0.76	0.44, 1.30	0.311

Sociodemographics			
Age	1.66	0.79, 3.46	0.180
Gender			
Girl	Ref.	Ref.	Ref.
Boy	0.54	0.19, 1.58	0.264
Race/Ethnicity			
African American	Ref.	Ref.	Ref.
Hispanic	1.09	0.35, 3.43	0.876
Another Race	0.99	0.20, 4.93	0.987
Sexual Orientation			
Gay/Lesbian/Bisexual/Other	Ref.	Ref.	Ref.
Heterosexual	0.83	0.27, 2.58	0.745

*DV* Dating Violence, *AOR * Adjusted odds ratio, *95% CI * 95% confidence interval, *Ref* Reference group

## Discussion

The purpose of this study was to examine the moderating effects of parental monitoring and closeness regarding the association between household dysfunction (HD) and dating violence (DV) perpetration among middle schoolers aged 11-14 years in Southeast Texas. Our study showed evidence of a significant relation between HD and lifetime DV perpetration; however, we did not find the parental protective factors to be significant individual moderators among our study population.

In accordance with existing literature, our results revealed a significant relationship between HD and lifetime DV perpetration [12–14]. We also found a larger effect size between HD and lifetime DV perpetration among our study population compared to these studies. Our more robust finding may be due to previous studies only examining two aspects of HD (i.e., witnessing interparental violence among parents and harsh parenting); we utilized a more comprehensive HD measure, which included several age-appropriate items that captured multiple aspects of HD. Furthermore, our findings also provided evidence regarding how greater diversity of HD exposures can promote engagement in risky behaviors such as DV perpetration as early as sixth grade. 

Prior literature has reported several parental protective factors (i.e., parental closeness and/or support, parental monitoring, and family functioning) to alleviate the consequences of ACEs, such as DV perpetration [6], violence perpetration [26], and cyber dating abuse [17]. Davis et al. [6] reported high social support significantly moderated the association between adolescents who were categorized under the lower exposure to family conflict and community violence class, leading to decreased odds of perpetrating physical or verbal DV. In addition, Gorman-Smith et al. [26] found that youth with better family functioning who were exposed to higher levels of community violence perpetrated less violence. Our findings differed from these studies as we specifically examined HD as the main ACE exposure with our outcome centered on lifetime DV perpetration among both sixth grade male and female adolescents. Furthermore, our study uniquely assessed parental monitoring and parental closeness as the moderators. Our results also differed from Peskin et al. [17] who reported that increased parental monitoring and parental closeness significantly reduced the odds of engaging in cyber DV perpetration among sixth graders. In contrast, our study specifically examined their moderating roles regarding the effect of HD on lifetime DV perpetration among sixth graders but found no moderation effect.

To date no studies have investigated the unique influences of parental monitoring and parental closeness regarding the association between HD and DV perpetration among middle school adolescents. Our results did not find these parental protective factors (i.e., parental monitoring and parental closeness) to be significant individual moderators. These findings could be due to several different reasons. First, our limited sample size may have affected our statistical power to identify significant moderating effects of parental monitoring and parental closeness regarding the association between HD and lifetime DV perpetration. Moreover, as the parental monitoring and parental closeness items appeared near the end of the survey, adolescents may have partially completed these measures because the survey was time-bound, which may have also contributed to the issue of missingness. Not all adolescents possess the same reading skills or speed to have completed the survey within the time frame. Given the limitations in statistical power due to the small analytic sample, the magnitude and direction of the moderation effects should be interpreted with caution. Notably, the interaction between parental monitoring and HD yielded an AOR of 0.76, suggesting a potential buffering effect, where higher levels of monitoring may attenuate the impact of HD on lifetime DV perpetration. In contrast, the interaction between parental closeness and HD had an AOR of 1.00, indicating no moderation effect. These patterns provide preliminary but meaningful insight into the potentially distinct roles of different parenting behaviors and underscore the need for future studies with larger, more representative samples to further investigate these associations.

Second, both parental monitoring and parental closeness measures did not include relevant aspects related to the adolescents’ DV behaviors or specific items about their dating history. Furthermore, adolescents may have been hesitant or sensitive to respond to specific items within the parental monitoring and parental closeness measures due to their relationship dynamics with their parents. Therefore, future research should explore other aspects of parental monitoring and closeness, such as including relevant items related to dating and disclosures of problematic DV behaviors among middle school aged adolescents [42].

### Strengths and limitations

To our knowledge, this is the first study that examined the individual moderating effects of parental monitoring and parental closeness regarding the association between HD and DV perpetration among sixth-grade adolescents in Southeast Texas. We also employed a newly developed, comprehensive age-appropriate HD measure to assess its impact on DV perpetration [34] whereas previous studies have primarily measured ACEs where HD was embedded within the overall paradigm and not assessed as an individual domain [9, 43, 44]. Although we did not find the parental factor moderators to be significant, we found the association between HD and lifetime DV perpetration to be significant, which demonstrated evidence regarding how early cumulative exposures to HD can initiate early risky behaviors such as DV perpetration, which can extend into adulthood. Thus, future studies should explore the relations among these multi-faceted constructs longitudinally using a larger and more diverse sample size to better understand the relation among these complex constructs in this population. 

Our study has several limitations. First, we had a relatively small sample size; thus, our results may have limited generalizability. We also utilized a cross-sectional study design; therefore, causal pathways between HD and DV perpetration and the effects of parental protective factors cannot be determined. We also relied on self-reported data, where adolescents may have been reluctant to respond to specific items due to issues related to societal stigma, fear, and judgment. In parallel with previous studies, we categorized adolescents who never dated or did not have a dating partner in the past 12 months as non-perpetrators [16, 33]. This study also did not account for household socioeconomic status (i.e., household family income or poverty level), which is a critical construct to control for when examining the relationship between HD and DV perpetration, as well as the influence of parental monitoring and closeness. Another limitation of this study is the presence of multicollinearity among some predictor variables, as shown by a mean VIF of 10.82. This may have increased the standard errors of the regression coefficients, making it harder to determine the unique contribution of each variable. We chose to keep all predictors in the model because they are important to the study’s aims, but we recognize that multicollinearity may affect the interpretability of the results. Future research with larger samples or different methods, like ridge regression or principal component analysis, could address this issue. Future studies should consider accounting for these variables and assess this association and the parental protective moderating effects among a larger and more diverse adolescents dating sample.

## Conclusion

This study investigated the moderating effects of parental monitoring and parental closeness regarding the relationship between HD and lifetime DV perpetration among sixth-grade middle schoolers in Southeast Texas. Our study findings underscored the significance of how early exposure to HD can initiate risky and violent behaviors when adolescents are just starting their romantic relationships. Furthermore, there is a need for future explorations regarding exploring the nuances of parent-child relationships, and implementing strategic interventions to promote positive parent-child relationships. Exploring these parental protective factors in a more detailed manner will help inform parent-focused interventions to decrease the immediate and long-term negative impact of HD and DV perpetration. 

## Supplementary Information

Supplementary Material 1.

Supplementary Material 2.

Supplementary Material 3.

Supplementary Material 4.

## Data Availability

Due to confidentiality issues, the data supporting the findings cannot be provided. However, interested parties may contact the corresponding author for additional information or clarification.

## Abbreviations

DV: Dating Violence
HD: Household Dysfunction
CDC: Centers for Disease Control and Prevention
YRBS: Youth Risk Behavior Surveillance System
ACEs: Adverse Childhood Experiences
(MY) Tech: Me and You Tech: A Socio-Ecological Solution to Teen Dating Violence for the Digital Age program
CADRI: Conflict in Adolescent Dating and Relation Inventory
