# Supplementary material for: Household dysfunction and dating violence perpetration: the moderating effects of parental monitoring and closeness among middle school adolescents in Southeast Texas
Source: BMC Public Health. 2025 Oct 3;25:3317. doi: 10.1186/s12889-025-24549-4 (PMC12495685; doi:10.1186/s12889-025-24549-4)
Supplement: Supplementary file 1 — Supplementary Material 1. [file 12889_2025_24549_MOESM1_ESM.docx]

##### **Supplement**

##### **Supplement 1: DV Perpetration Measures**

| **CADRI DV Subscales** | **Items** |
| --- | --- |
| **Instruction:** The following questions ask about things that you may have ever done toward a boyfriend or girlfriend (someone that you have dated, gone out with, gone steady with). Please mark YES or NO for each question. Remember, all of your answers will be kept private, and no one will know your answers. | |
| Physical | 1. I threw something at him/her. 2. I kicked, hit, or punched him/her. 3. I slapped him/her or pulled his/her hair. 4. I pushed, shoved, or shook him/her. |
| Threatening | 1. I destroyed or threatened to destroy something he/she valued. 2. I tried to frighten him/her on purpose. 3. I threatened to hurt him/her. 4. I threatened to hit him/her or throw something at him/her. |
| Sexual | 1. I kissed him/her when he/she didn’t want me to. |
| Psychological | 1. I did something to make him/her jealous. 2. I brought up something bad that he/she had done in the past. 3. I said things just to make him/her angry. 4. I spoke to him/her in a hostile or mean tone of voice. 5. I insulted him/her with put-downs. 6. I made fun of him/her in front of others. 7. I kept track of who he/she was with and where he/she was. 8. I blamed him/her for the problem. 9. I accused him/her of flirting with another girl/guy. 10. I threatened to end the relationship. 11. I tried to turn my friends against him/her. 12. I said things to his friends about him/her to turn them against him/her. 13. I spread rumors about him/her. |
| **Digital DV Perpetration Measure Adapted from Zweig et al. (2014) and Picard (2007)** | |
| Digital | 1. I posted embarrassing photos or other images of him/her online. 2. I sent threatening text messages to him/her. 3. I used his/her social networking account (Facebook, Twitter, Instagram, etc.) without his/her permission. 4. I wrote nasty things about him/her on my profile page/timeline (on Facebook, Twitter, Instagram, etc.). 5. I sent him/her so many messages (like texts, emails, chats) that it made him/her feel unsafe. 6. I sent him/her text messages to check up on him/her (where are you, what are you doing, who are you with). 7. I spread rumors about him/her using a cell phone, email, IM, web chat, or social networking site (on Facebook, Twitter, Instagram, etc.). 8. I used information from his/her social networking profile/page to harass him/her or put him/her down. 9. I made him/her afraid when he/she did not respond to my phone call, text, posting on social networking page, IM, et. 10. I threatened to harm him/her physically through a cell phone, text message, social networking page, etc. 11. I sent him/her sexual photos or naked photos of me that I knew he/she did not want. 12. I pressured him/her to send a sexual or naked photo of him/herself. |

Note: DV = Dating Violence.
