# Supplementary material for: Household dysfunction and dating violence perpetration: the moderating effects of parental monitoring and closeness among middle school adolescents in Southeast Texas
Source: BMC Public Health. 2025 Oct 3;25:3317. doi: 10.1186/s12889-025-24549-4 (PMC12495685; doi:10.1186/s12889-025-24549-4)
Supplement: Supplementary file 2 — Supplementary Material 2. [file 12889_2025_24549_MOESM2_ESM.docx]

##### **Supplement**

##### **Supplement 4: Parental Closeness Measure**

| **Parental Closeness Measure** |
| --- |
| **Instruction:** The following statements are some opinions about parent(s) or caregiver(s). Some people your age agree and others disagree with these statements. Please choose the response that matches your own opinion about your parent(s)/guardian(s). |
| 1. My parent/caregiver often asks about what I am doing in school. |
| 1. My parent/caregiver gives me the right amount of affection. |
| 1. One of the worst things that could happen to me would be to find out that I let my parent/caregiver down. |
| 1. My parent/caregiver is usually proud of me when I finish something at which I’ve worked hard. |
| 1. My parent/caregiver trusts me. |
| 1. I am closer to my parent/caregiver than are a lot of kids my age. |
