# Supplementary material for: Household dysfunction and dating violence perpetration: the moderating effects of parental monitoring and closeness among middle school adolescents in Southeast Texas
Source: BMC Public Health. 2025 Oct 3;25:3317. doi: 10.1186/s12889-025-24549-4 (PMC12495685; doi:10.1186/s12889-025-24549-4)
Supplement: Supplementary file 3 — Supplementary Material 3. [file 12889_2025_24549_MOESM3_ESM.docx]

##### **Supplement**

##### **Supplement 3: Parental Monitoring Measure**

| **Parental Monitoring Measure** |
| --- |
| **Instruction:** Please continue to think about your parent or caregiver for the next questions |
| 1. How much does your parent or caregiver know about who your friends really are? |
| 1. How much does your parent or caregiver know about where you are most afternoons after school? |
| 1. How much does your parent or caregiver really know about how you spend your money? |
| 1. How much does your parent or caregiver really know about where you go at night? |
| 1. How much does your parent or caregiver really know about how you spend your free time? |
