# Supplementary material for: Household dysfunction and dating violence perpetration: the moderating effects of parental monitoring and closeness among middle school adolescents in Southeast Texas
Source: BMC Public Health. 2025 Oct 3;25:3317. doi: 10.1186/s12889-025-24549-4 (PMC12495685; doi:10.1186/s12889-025-24549-4)
Supplement: Supplementary file 4 — Supplementary Material 4. [file 12889_2025_24549_MOESM4_ESM.docx]

# **Supplement**

##### **Supplement 2: HD Measure**

| **HD Measure** |
| --- |
| 1. Do you live with anyone who is sad the majority of the time? |
| 1. Do you live with anyone who drinks too much? |
| 1. Do you live with anyone who abuses drugs or their medications? |
| 1. Do you live with anyone who went to jail or is currently in jail? |
| 1. Are your parents separated or divorced? |
| 1. Do you live with anyone who says mean things or hurts other members in your house? |
| 1. Have you lived with a parent or guardian who died? |
| 1. Is your family having problems with stable housing? (For example, not having a permanent place to live, having to move often, or having to live with multiple family members). |
| 1. Do you often worry that you do not have enough food to eat at home? |
| 1. Do you live with anyone who has a serious physical illness or disability? |

Note: HD = Household Dysfunction.
